# Critical Review on the Anti-Tumor Activity of Bioactive Compounds from Edible and Medicinal Mushrooms over the Last Five Years

**DOI:** 10.3390/nu17111887

**Published:** 2025-05-30

**Authors:** Sandra Górska-Jakubowska, Yingzi Wu, Jadwiga Turło, Baojun Xu

**Affiliations:** 1 Department of Drug Technology and Pharmaceutical Biotechnology, Medical University of Warsaw, 1 Banacha Str., 02-097 Warsaw, Poland; sandra.gorska@wum.edu.pl (S.G.-J.); jadwiga.turlo@wum.edu.pl (J.T.); 2 Food Science and Technology Program, Department of Life Sciences, Beijing Normal-Hong Kong Baptist University, Zhuhai 519087, China; wuyingzi@uic.edu.cn; 3 School of Chinese Medicine, Hong Kong Baptist University, Hong Kong, China

**Keywords:** mushroom, bioactive compounds, anti-cancer effect, mechanism

## Abstract

In recent years, the incidence rate of cancer has been on the rise all over the world, and the age of cancer patients has shown a younger trend, which seriously endangers patients’ health. Edible/medicinal mushrooms have not only become a new source of nutritional supplements but have also emerged as a promising adjunct to conventional medicine, either by directly or indirectly killing tumor cells and enhancing immunity, or through their use in conjunction with modern cancer therapies to enhance their efficacy or reduce their side-effects, improving patients’ quality of life. Although the anti-cancer potential of edible and medicinal mushrooms has been widely studied in the past, this review focuses on the most recent literature from the last five years, providing an up-to-date and comprehensive summary of the current findings. In this review, we aim to analyze the anti-cancer effects of edible/medicinal mushrooms, including *Schizophyllum commune*, *Trametes versicolor*, *Grifola frondosa*, *Ganoderma lucidum*, *Lentinula edodes*, *Laetiporus sulphureus*, *Boletus edulis*, and *Phellinus igniarius*, as well as their potential anti-cancer mechanisms, providing strong theoretical support for the further development of edible/medicinal mushroom anti-cancer products.

## 1. Introduction

Cancer remains the second leading cause of death globally, second only to cardiovascular disease. In 2024, the United States is predicted to have 2,001,140 new cancer cases and 611,720 cancer-related deaths. During 2015–2019, the incidence rate of breast cancer, pancreatic cancer, and uterine body cancer gradually increased every year, while the incidence rate of prostate cancer, liver cancer (female), kidney cancer, HPV-related oral cancer, and melanoma increased by 2–3% every year. Among young people, the incidence rate of cervical cancer (30–44 years old) and colorectal cancer (<55 years old) has also been increasing every year. Moreover, cancer poses a significant threat to public health worldwide, especially in poor and developing countries. This situation deserves our focused attention [1].

At present, the conventional methods for treating cancer in clinical practice include surgery, chemotherapy, immunotherapy, radiotherapy, targeted therapy, and endocrine therapy. Although advanced treatment methods have slowed down the disease progression of patients to some extent in the past few decades, cancer cells may develop a acquired resistance, thereby reducing the tumor’s response to conventional treatments. In addition, these treatments may lead to serious adverse reactions, such as bone marrow suppression, gastrointestinal diseases, hemorrhagic cystitis, and hair loss, as well as cardiovascular and neurotoxicity, thereby limiting the patient’s quality of life. In addition, new cancer treatment methods, such as targeted therapy and immunotherapy, are expensive and impose heavy economic and psychological burdens on patients [2].

Natural products, preferably those consumed in diets, offer a promising supportive strategy in cancer therapy. In the evaluation of complementary and integrated methods, the combination of conventional medicine and complementary activities, including the use of various products such as the extracts of mushrooms or medicinal herbs, vitamins, nutritional supplements, and probiotics, has shown promising results [3,4,5]. Edible and medicinal mushrooms are consumed worldwide, both as foods and as nutritional supplements. While many of these mushrooms, such as *G. lucidum* and *L. edode*, are commonly included in the diet for their culinary value, their bioactive compounds, including polysaccharides (especially β-glucan), polysaccharide peptides, glycoprotein fractions, and triterpene, etc., are increasingly being investigated for their therapeutic potential. These compounds have shown promising effects, including immune modulation, anti-inflammatory properties, and anti-cancer activity, leading to their use in nutritional supplements and functional foods. Some mushrooms have even shown potential for development into pharmaceutical drugs for specific therapeutic purposes, especially in cancer prevention and in adjunct cancer therapies [6,7,8,9]. Multiple studies have also shown that the bioactive compounds of mushrooms negatively regulate key molecular targets involved in processes such as cell proliferation, survival, and angiogenesis. These anti-cancer effects may be linked to regulating signaling pathways such as the PI3K/AKT signaling pathway [10], the EGFR/Raf signaling pathway [11], the mTOR/AMPK signaling pathway [12], the P53 signaling pathway [13], the Dectin-1/Syk/NF-κB signaling pathway [14], and the TLR4/NF/κ B signaling pathway [15].

**Figure 1:**
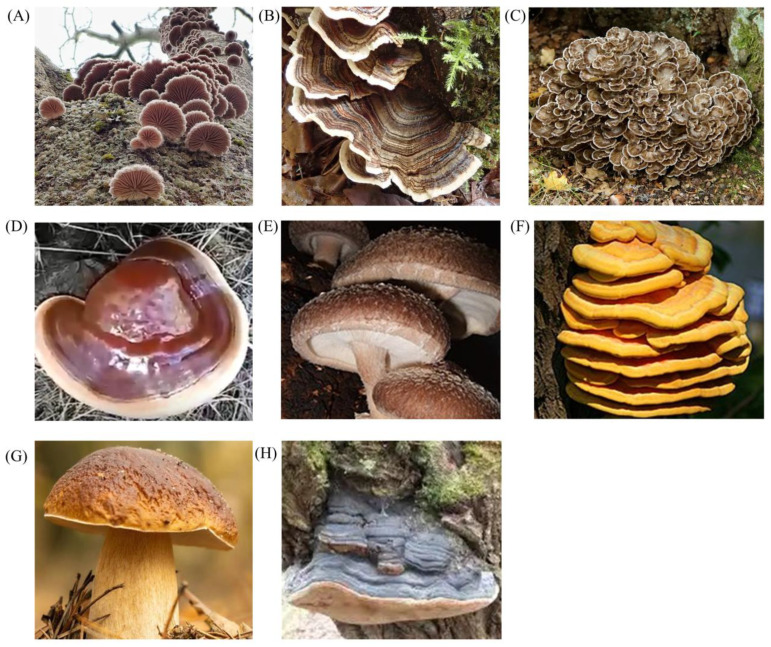
Eight edible/medicinal mushrooms mentioned in the article: (**A**) *S. commune*; (**B**) *T. versicolor*; (**C**) *G. frondosa*; (**D**) *G. lucidum*; (**E**) *L. edodes*; (**F**) *L. sulphureus*; (**G**) *B. edulis*; (**H**) *P. igniarius*. All of these images were sourced from Google.

Although the anti-cancer properties of edible and medicinal mushrooms have been widely explored in the scientific literature, this review stands out by offering the most current and comprehensive synthesis of the research published in the last five years to provide theoretical support for exploring the potential of more mushroom bioactive compounds in clinical practice. It focuses specifically on the anti-tumor potential of edible/medicinal mushrooms, including *Schizophyllum commune* (Fries), *Trametes versicolor* (Linnaeus) Lloyd, *Grifola frondosa* (Dickson) Gray, *Ganoderma lucidum* (Fries) P. Karsten, *Lentinula edodes* (Berkeley) Pegler, *Laetiporus sulphureus* (Bulliard) Murrill, *Boletus edulis* Bulliard, and *Phellinus igniarius* (Linnaeus) Quélet (Figure 1). The selection of these nine mushrooms for this review was based on the fact that they have both been widely recognized for their potential health benefits and extensively studied in the context of anti-tumor activity. We report on a variety of insights into the mechanisms by which their bioactive compounds exert anti-tumor effects on different types of cancer, providing strong theoretical support for the further development of edible/medicinal mushroom anti-cancer products. This review also provides strong theoretical support for developing products that cater to consumers seeking natural solutions for overall healthcare or cancer prevention.

## 2. Methodology

### 2.1. Information Sources and Search Strategy

This narrative review was conducted in three main stages: a literature search, a screening of the abstracts and full-texts, and a discussion of the results. To identify relevant studies, we searched the widely used PubMed (https://pubmed.ncbi.nlm.nih.gov/, accessed on 1 March 2025) and Google Scholar (https://scholar.google.com/, accessed on 1 March 2025) databases. The final search was completed in February 2025, focusing on international English-language articles, online reports, and e-books. The search employed a combination of keywords, including *Schizophyllum commune*, *Trametes versicolor*, *Grifola frondosa*, *Ganoderma lucidum*, *Lentinula edodes*, *Laetiporus sulphureus*, *Boletus edulis*, *Phellinus igniarius*, and cancer, along with related terms such as characteristics, habitats, bioactive compounds, molecular mechanisms, clinical trials, bioavailability, therapeutic uses, challenges, and regulations, as well as terms like tumor, oncology, and neoplasms. After the search was conducted, the abstracts were reviewed to ensure that they were relevant to the topic. Duplicates were removed, and the remaining abstracts were carefully evaluated against the inclusion criteria, which focused on studies analyzing the anti-cancer effects of bioactive compounds in edible/medicinal mushrooms in conjunction with the aforementioned terms. Based on this screening, a summary and synthesis of the relevant research were performed to provide an integrated narrative analysis [16,17].

### 2.2. Eligibility and Exclusion Criteria

All international studies were considered for inclusion. The eligibility requirements for this study were as follows: (1) articles must be available in English; (2) articles must be published between 1 January 2020 and 1 February 2025; (3) the anti-cancer effects of eight edible/medicinal mushrooms; (4) extraction methods of bioactive compounds in eight edible/medicinal mushrooms; (5) in vitro and in vivo studies evaluating the effects of eight mushroom extracts on various cancer types; (6) mechanistic studies investigating the anti-cancer properties of eight mushroom bioactive compounds in vitro and in vivo. Relevant articles not found by the primary search strategy were still included in the reference lists, which were also thoroughly reviewed.

**Figure 2:**
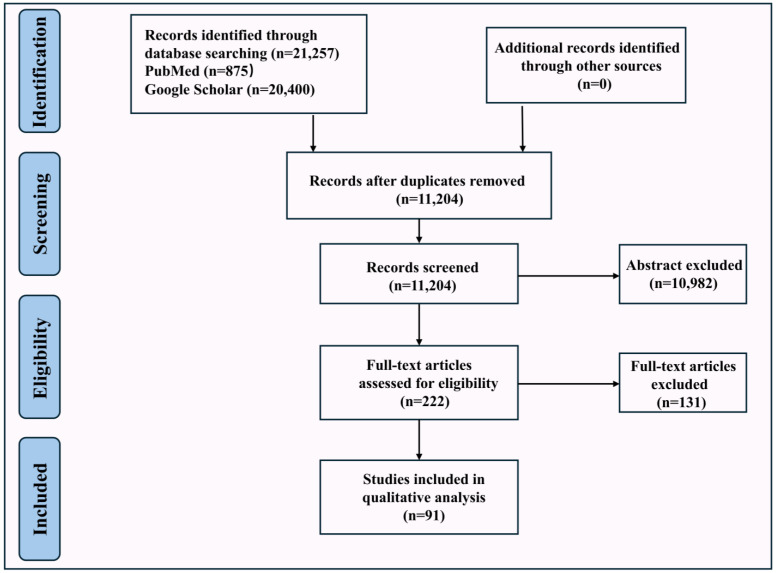
Flowchart of the literature search and screening method.

The exclusion criteria applied in this review were as follows: (1) non-systematic and narrative reviews; (2) articles published in languages other than English; (3) editorial materials; (4) studies with limited relevance to the topic; (5) non-peer-reviewed literature; (6) repetition of studies previously published elsewhere; (7) studies that did not provide detailed methods, original data, or results; (8) studies for which the full text was not available. These criteria, as well as inclusion criteria, are described in the flow chart in Figure 2.

## 3. Summary of Research on the Anti-Cancer Properties of Active Compounds in Edible/Medicinal Mushrooms

### 3.1. Schizophyllum commune

#### 3.1.1. Occurrence and Ecology

*Schizophyllum commune* (*S. commune*) is a widely distributed saprotrophic fungus, found on all continents except Antarctica. It prefers humid, temperate climates but can thrive in various environments, from tropical rainforests to cooler, temperate forests. This fungus primarily grows on dead hardwoods such as oak, beech, and birch, but it can also colonize coniferous wood and other plant materials. *S. commune* plays a crucial role in the decomposition of lignin, one of the primary components of wood, which is vital for nutrient cycling in forest ecosystems. Its lignin-degrading ability is essential for mineralization processes that support forest health and productivity. This fungus is frequently observed in urban environments, colonizing wooden structures such as fences and outdoor furniture. Its fan-shaped, leathery fruiting bodies can appear throughout the year under favorable moisture conditions. *S. commune* is a perennial fungus, with its fruiting bodies often persisting for several seasons, making it easily recognizable in various habitats [18].

#### 3.1.2. Anti-Cancer Properties of Bioactive Components from *S. commune*

*S. commune* contains a variety of active ingredients, including polysaccharides, polyphenols, flavonoids, and terpenoids, with polysaccharides being the primary bioactive components. These components exhibit a range of beneficial effects, such as anti-cancer, anti-bacterial, anti-inflammatory, immune-regulatory, and hypoglycemic effects. Notably, the fruiting body polysaccharides of *S. commune* (SCFP) are heteropolysaccharides, with a molecular weight of 290.92 kDa, primarily composed of mannose, galactose, and glucose. In vitro studies have shown that SCFP significantly inhibits the proliferation of glioma in a concentration-dependent manner. The anti-cancer mechanism of SCFP may be related modulating the PI3K/AKT signaling pathway and inducing apoptosis, thereby suppressing cancer cells’ growth [19].

SCP-1 is a highly branched mucomannan from the fruiting body of *S. commune*. Its molecular weight is 15.1 kDa, composed of 1,3-linked β-D-glucose/α-D-glucose and 1,2-linked α-D-mannose, and it has an O-6 branched chain. It has a different structure from traditional *Schizophyllum* polysaccharides. Research has shown that SCP-1 has a significant growth-inhibitory effect on A549 lung cancer cells; it can arrest the cell cycle in the S phase; and it induced apoptosis in A549 cells through the Bax/Bcl-2 and PI3K/Akt/mTOR signaling pathways [10]. Further research needs to focus on the relationship between its structure and its anti-tumor activity and compare the differences in its anti-tumor activity from polysaccharides from *S. commune* in order to better understand its anti-cancer activity.

Another study indicated that *S. commune* extraction, which included terpenoids, flavonoids, and alkaloids, could inhibit proliferation of different kinds of cancer cell lines, such as HeLa, MCF7, T47D, and WiDr [20]. It is imperative to conduct further research in order to accurately ascertain its primary bioactive components.

Gold nanoparticles have excellent properties, including size regulation, stability, and biocompatibility, which can be used for the treatment and diagnosis of cancer. Gold nanoparticles (AuNPs) were mycologically synthesized using *S. commune* fungi. The research results indicate that AuNPs significantly reduce the cell viability of A549 cancer cells in a dose-dependent manner. In addition, AuNPs were observed to significantly increase intracellular ROS in A549 cancer cells [21].

Schizophyllan (SPG), derived from the fungus *S. commune*, features a β-(1→3)-glucan backbone and has garnered attention in recent years for its broad biomedical potential. SPG has significant immunomodulatory effects and can enhance the body’s innate and adaptive immune responses. Research has shown that SPG can activate intracellular signaling pathways and promote the secretion of cytokines and chemokines by binding to receptors on the surface of immune cells such as dectin-1, thereby enhancing the activity and function of immune cells and indirectly exerting anti-tumor effects. In addition, SPG can also be used as a drug carrier for delivering anti-tumor agents. Its unique triple helix structure and good biocompatibility enable it to effectively bind to drug molecules, achieving targeted delivery and controlled release of anti-tumor drugs [22].

An in vivo study indicated that SCFP suppressed the growth of glioma. In this study, 10 of the 30 nude mice were randomly assigned to the normal group, and the remaining 20 nude mice were used to construct a U251 cell line-derived xenograft tumor model. SCFP treatment has been found to increase the relative abundance of beneficial bacteria and restore the gut microbiota structure of tumor-bearing mice to levels observed in tumor-free mice [19].

The studies on *S. commune*’s bioactive components have shown promising results but there are some limitations that need to be considered. First, the studies lack sufficient clinical data to support the findings from in vitro and in vivo experiments. Second, some studies may have small sample sizes, which can affect the reliability and generalizability of the results. Third, the anti-cancer effects and mechanisms of action of these bioactive components may vary depending on factors such as the source, extraction method, and preparation of the mushroom extracts. More research is needed to standardize these processes and ensure consistent results. In addition, the long-term safety and efficacy of using *S. commune* extracts or its bioactive components for cancer treatment have not been fully established

Despite these limitations, the existing studies provide valuable insights into the potential anti-cancer properties of *S. commune* and its bioactive components and highlight the need for further research to fully understand their potential applications in cancer therapy.

### 3.2. Trametes versicolor

#### 3.2.1. Occurrence and Ecology

*Trametes versicolor* (*T. versicolor*), commonly known as turkey tail, is a saprotrophic fungus widely distributed across various habitats globally. *T. versicolor* is also known as *Coriolus versicolor* (Linnaeus) Quélet [23]. It primarily colonizes dead hardwoods such as oak, beech, and birch, though it can also grow on coniferous wood, demonstrating its adaptability to different substrates. *T. versicolor* is particularly abundant in temperate forests, where it plays a crucial role in the decomposition of lignin and cellulose, contributing to the nutrient cycle within forest ecosystems. The fruiting bodies of *T. versicolor* are highly distinctive due to their multicolored, zoned appearance, resembling the tail feathers of a turkey, which gives the species its common name. These fan-shaped fruiting bodies grow in dense clusters and can be found throughout the year, although they are most prominent during moist and cooler months, making them easily identifiable in their natural habitats [24].

#### 3.2.2. Anti-Cancer Properties of Bioactive Components from *T. versicolor*

*T. versicolor* is a rich source of various bioactive compounds, including polysaccharides, triterpenes, phenols, and proteins, all of which have significant medicinal potential. Among these, the most notable are polysaccharide-K (PSK) and polysaccharide peptides (PSP). Numerous studies have shown that PSP and PSK, whether administered orally, intravenously, or intraperitoneally, have strong and widespread anti-tumor activity and are generally considered non-toxic and have no adverse reactions. Previous experiments have suggested that PSK showed anti-tumor activity against different kinds of malignant tumors, including leukemia, liver cancer, ovarian cancer, breast cancer, colorectal cancer, and other cancer cells [23,25]. As a commercial product, the main sources of these PSPs are China and Japan, which produce them from the strains of “COV-1” (PSP in China) and “CM-101” polysaccharide K (PSP Krestin or PSK in Japan), respectively. Both products have been approved as medicines primarily as adjuvants in cancer therapy [23].

In a study aimed at evaluating the cytotoxic effects of ethanol extracts from the fruiting body and mycelium of *T. versicolor* on A375 and SK-MEL-5 human melanoma cell lines, it was noted that the ethanol extract of *T. versicolor* mycelium had a significant inhibitory effect on malignant melanoma cells, and the cytotoxicity of *T. versicolor* mycelium extract on A375 and SK-MEL-5 cell lines was stronger than that of the fruiting body extract. *T. versicolor* mycelium extract induced apoptotic cell death and poly (ADP ribose) polymerase cleavage, increased the expression of autophagy-related marker LC3-II, increased the expression of major histocompatibility complex II and programmed death ligand receptor, and suppressed cell migration in SK-MEL-5 cells. Therefore, *T. versicolor* mycelium extract deserves further investigation [26].

Research has suggested that PSP may serve as a prophylactic and therapeutic agent against colorectal cancer (CRC) by down-regulating the programmed cell-death ligand 1 (PD-L1) and epidermal growth factor receptor (EGFR) signaling pathways [11]. In this study, PSP not only reduced the expression of EGFR but also promoted the death of colorectal cancer cells through the EGFR pathway in colorectal cancer cells, indicating that PSP has significant potential as a PD-L1 inhibitor and an immunological supplement in the treatment of CRC.

Musarin is a novel 12 kDa polysaccharide peptide isolated from *T. versicolor*. Research has indicated that musarin significantly inhibits the proliferation of different colorectal cancer cells in a dose-dependent manner, and it particularly inhibits the proliferation of CD24^+^ CD44^+^ HT29 cells with CSC characteristics. In addition, musarin exhibited tyrosine kinase inhibitory activity, especially against the activation of EGFR, and regulates the up-regulation and down-regulation of genes in the EGFR-Ras signaling pathway; this may be one of the mechanisms by which it inhibits the proliferation of colorectal cancer stem cells. This was the first accurate report on the molecular mechanism of the inhibitory effect of *T. versicolor*-derived polysaccharide peptides on colorectal cancer [27].

Scholars have extracted five steroid compounds from *T. versicolor*, including 5 α, 8 α-epidioxygeergosta-6.22-dien-3 β-ol (compound 1), (22E, 24R)-ergosta-7.22-diene-3 β, 5 α, 6 β-triol (compound 2), 9,19-cyclolanostane-3,29-diol (compound 3), ergosta-7.22-dien-3-acetate (compound 4), and ergosta-8 (14), 22-dien-3 β, 5 α, 6 β, and 7 α-control (compound 5). Among them, compound 1 showed the strongest inhibitory effect on most cancer cell lines and was effective on most of the 58 human cancer cell lines, especially non-small-cell lung cancer, melanoma, and colon cancer cell lines [28].

Furthermore, recent studies have demonstrated the potential of selenium-enriched exopolysaccharides (Se-cEPS) synthesized by *T. versicolor* in submerged culture. By using selenourea and sodium selenite as selenium sources, researchers achieved significant Se accumulation in crude exopolysaccharides. The selenium-containing fractions, composed of polysaccharides and proteins (proteoglycans), exhibited notable biological properties, including strong antioxidant activity (DPPH radical scavenging, Fe^2+^ chelation) and broad-spectrum anti-bacterial effects, especially against Gram-positive strains. While their direct anti-cancer effects remain to be explored in depth, the established antioxidative and immunomodulatory potential of Se-cEPS underscores their relevance in cancer prevention strategies through oxidative stress modulation and microbiota balance maintenance [29].

The small molecules from *T. versicolor* (SMCV) not only have indirect anti-cancer effects by inhibiting TNF-α-induced MMP-3 production in glioblastoma T98G cells but also directly reduce the invasive ability of malignant cells such as T98G, A549, and MDA-MB-231 cells [30]. This dual mechanism of immune modulation and direct cytotoxicity makes *T. versicolor* a potent candidate for cancer therapy.

The tumor microenvironment plays a crucial role in cancer progression, and *T. versicolor* has been shown to modulate this environment in favor of anti-tumor activity. It can shift the polarization of tumor-associated macrophages (TAMs) from the pro-tumorigenic M2 phenotype to the anti-tumorigenic M1 phenotype. This shift is characterized by the up-regulation of pro-inflammatory cytokines such as IL-6 and TNF-α, which have anti-tumor properties, and the down-regulation of immunosuppressive cytokines such as IL-10 and TGF-β [31]. By altering the balance of cytokines and immune cells within the tumor microenvironment, *T. versicolor* can create a less favorable environment for tumor growth and metastasis.

In addition to its standalone anti-cancer effects, *T. versicolor* has been shown to enhance the efficacy of conventional cancer therapies such as chemotherapy and radiotherapy. For instance, when combined with chemotherapeutic agents, PSP can potentiate their cytotoxic effects on cancer cells, leading to greater tumor suppression. This synergistic effect is attributed to the ability of *T. versicolor* to sensitize cancer cells to the cytotoxic effects of chemotherapy, while simultaneously mitigating the immunosuppressive side-effects of these treatments [25].

Moreover, when combined with LY294002, an inhibitor of the phosphatidylinositol-3-kinase (PI3K) signaling pathway, *T. versicolor* extract promoted cancer cell treatment (HeLa and MCF-7 cells), potentially leading to a novel therapeutic approach [32]. In this study, compared to usage of either agent alone, the combination of *T. versicolor* extract and LY294002 demonstrated enhanced efficacy in inducing G0/G1 cell cycle arrest, promoting apoptosis, suppressing cell migration and invasion, and reducing the expression of phosphorylated PI3K. These results underscore the potential of this combination therapy as a novel strategy for cancer treatment, emphasizing the importance of targeting the PI3K pathway in enhancing therapeutic outcomes. Future research should focus on elucidating the detailed molecular pathways involved in these mechanisms and exploring the potential of *T. versicolor* in combination with other natural compounds or conventional treatments to develop more effective cancer therapies.

In summary, active components from *T. versicolor* have excellent anti-cancer activity with low toxicity, which indicated that *T. versicolor* has the potential for further development. However, research in the past five years has mainly focused on in vitro studies, and in the future, emphasis should be placed on in vivo experiments and even clinical trials to further confirm its anti-cancer ability and mechanism of action.

### 3.3. Grifola frondosa

#### 3.3.1. Occurrence and Ecology

*Grifola frondosa* (*G. frondosa)*, *a*, commonly known as maitake, is a polypore mushroom that primarily grows at the base of oak trees and other hardwoods. It naturally occurs in temperate regions across Asia, Europe, and North America. *G. frondosa* prefers cool, moist forest environments, where it is typically found in late summer and autumn. The fruiting bodies of this fungus form branched structures that can reach impressive sizes, sometimes weighing several kilograms. *G. frondosa* plays a crucial role in forest ecosystems by decomposing dead wood, contributing to nutrient recycling in the soil. In some regions, this mushroom is also cultivated commercially due to its culinary and medicinal value [33].

#### 3.3.2. Anti-Cancer Properties of Bioactive Components from *G. frondosa*

There are many bioactive substances in *G. frondosa*, such as β- glucan and protein units, including D component, X component, Grilofan, and SX component, which have strong anti-proliferative and immune-regulatory effects. Numerous studies have suggested that it has the ability to activate key immune cells, especially macrophages, natural killer cells (NK), and cytotoxic T cells, which play a crucial role in immune defense and direct tumor cell destruction. In addition, its glucan stimulates the production of cytokines such as interleukin-1 and interleukin-2, which are key mediators of immune responses [34].

GFPBW1 is a novel soluble 300 kDa homogeneous β-glucan derived from the fruiting body of *G. frondosa*. It activates macrophages by targeting the Dectin-1/Syk/NF-κB signaling pathway, exerting anti-tumor effects [14].

A polysaccharide (GFP-A) from *G. frondosa* could significantly protect the thymus and spleen, inhibit the growth of S180 tumor cells by inducing cell cycle arrest in the G1 phase and apoptosis, enhance the killing activity of NK cells, the phagocytic ability of macrophages, and the proliferation activity of splenic lymphocytes, and promote the secretion of cytokines such as TNF-α, IL-2, and IFN-γ to enhance immune function in vivo [35]. Another polysaccharide, designated as GFP1, has been demonstrated to inhibit the proliferation of the lung cancer cell line H1975 by inducing apoptosis. GFP1 increased intracellular reactive oxygen species (ROS) levels, decreased mitochondrial membrane potential, and up-regulated the caspase-3 expression of caspase-8 and caspase-9. Furthermore, it promotes the phosphorylation of p65, which, in turn, activates the NF-κB signaling pathway. This activation leads to the up-regulation of various pro-inflammatory cytokines and chemokines, thereby exerting immunomodulatory effects that can enhance the immune response against pathogens or cancer cells [36].

Se-LMW-GFP is a type of Se-modified low-molecular-weight *G. frondosa* polysaccharides (LMW-GFP). Studies have shown that treatment with Se-LMW-GFP leads to significant changes in cell morphology, increased apoptosis rate, decreased mitochondrial membrane potential, increased ROS levels, and cell cycle arrest in the G1 phase. The expression of related apoptotic proteins is up-regulated, indicating that Se-LMW-GFP can induce apoptosis in gastric cancer cells through exogenous death receptor pathways and endogenous mitochondrial pathways. Therefore, Se-LMW-GFP has the potential to serve as a novel functional health food and selenium supplement, and it is expected to be used in combination therapies for malignant tumors in the future [37].

*G. frondosa* polysaccharide–protein complex (PPC) is a polymer which consists of polysaccharides and proteins/peptides linked by covalent bonds. GFG-4 and GFG-100 were isolated from *G. frondosa* via water extraction at 4 °C and 100 °C. There is an in vivo study that evaluates the anti-cancer effect of GFG-4 and GFG-100. Male BALB/c mice (5–6 weeks, 20 ± 2 g) were used, and a H22 cell line xenograft tumor model was set up. The mice were randomly divided into seven groups (eight mice/group), including a blank control group, model group, a cyclophospha-mide (CTX)- positive group, a 100 mg/kg-GFG-4 group, a 300 mg/kg-GFG-4 group, a 100 mg/kg-GFG-100 group, and a 300 mg/kg-GFG-100 group. The results indicated that GFG-4 has the potential to inhibit the growth of hepatocellular carcinoma by activating the TLR4/NF-κB signaling pathway and regulating gut microbiota (increasing the abundance of norank_f__*Muribaculaceae* and *Bacillus*, while decreasing the abundance of lactic acid bacteria) in H22 cell line xenograft tumors in nude mice [15].

Additionally, studies analyzed the adjuvant effect of GFPBW1 using the OVA antigen and B16-OVA tumor model in mice. Female C57BL/6 mice were randomly divided into four groups (n = 5), including a control group, a 10 μg-OVAgroup, and OVA in combination with GFPBW1 (50 or 300 μg per mouse) groups. The results show that it significantly and dose-dependently increases the titers of OVA-specific antibodies of various subtypes (including IgG1, IgG2a, IgG2b, and IgG3), indicating its potential as an adjuvant for both Th1 and Th2 immune responses. Mice immunized with OVA plus GFPBW1 showed no significant pathological damage in major organs or at injection sites, and no abnormalities were found in any hematological parameters [14].

In summary, polysaccharides from *G. frondosa* have shown outstanding anti-cancer activity with various mechanisms of action. Se modification can improve its anti-cancer effect. However, there is still a lack of research on bioavailability and clinical aspects. For example, β-glucans have low oral bioavailability, which limits their systemic therapeutic potential. Given this, biomaterials can be used to solve this problem. In the future, researchers can actively supplement this substance and lay a more comprehensive foundation for it to become an effective anti-cancer drug.

### 3.4. Ganoderma lucidum

#### 3.4.1. Occurrence and Ecology

*Ganoderma lucidum* (*G. frondosa*), commonly known as Reishi or Lingzhi, is a medicinal and edible fungus belonging to the phylum *Basidiomycota*. It is predominantly found across Europe, America, Africa, and Asia, particularly in tropical, subtropical, and temperate regions. This fungus typically thrives in damp, low-light forest environments, growing on decaying wood or the roots of trees, and can be harvested throughout the year [38].

#### 3.4.2. Anti-Cancer Properties of Bioactive Components from *G. lucidum*

Modern research has identified a variety of health-promoting active ingredients in *G. lucidum*, such as polysaccharides, triterpenes, steroids, amino acids, nucleosides, alkaloids, and trace elements. These active components have been confirmed by various studies to possess immunomodulatory [39], anti-inflammatory [40], and anti-cancer effects [41,42]. The main metabolites with anti-tumor activity in this edible fungus are polysaccharides and triterpenoids. Among the polysaccharides, β-D-glucans show the main anti-tumor properties, especially β-1-3 and β-1-6-D-glucans, because they can activate effector cells such as macrophages, T helper cells, and natural killer cells, which bind to leukocytes and serum-specific proteins. This increases the production of cytokines such as interleukins, tumor necrosis factor α (TNFα), interferons (IFN), and nitric oxide (NO) [43]. In Japan and China, extracts of *G. frondosa* are often used as an adjuvant therapy for radiotherapy or chemotherapy in cancer patients. Research has shown that extracts from *G. frondosa* can improve the survival rate of patients and enhance the activity of immune cells. In addition, it can counteract the immunosuppressive effects of traditional anti-cancer therapies and alleviate cancer-related symptoms such as fatigue, loss of appetite, vomiting, and pain, thereby significantly improving the quality of life of cancer patients [44].

*G. lucidum* polysaccharides (GLPs) were able to restrain the proliferation, migration, and invasion of oral squamous cell carcinoma (OSCC) cells while down-regulating the expression of CSC and EMT markers, which may help to enhance the efficacy of traditional treatments and improve the quality of life of OSCC patients [42]. Fudan-Yueyang-*G. lucidum* (FYGL) is a proteoglycan extracted from *G. lucidum.* It has been reported that FYGL can selectively induce apoptosis of pancreatic cancer cells by regulating ROS and inhibiting autophagy, providing a new strategy for the treatment of pancreatic cancer [45]. In addition, as a natural product, *G. lucidum* polysaccharide (GLP) has the potential to reduce the side-effects of chemotherapy drugs and improve the therapeutic effect. Recent studies have confirmed that GLP could enhance the sensitivity of prostate cancer cells to flutamide and docetaxel. When GLP was used in combination with flutamide and docetaxel, it could significantly down-regulate the expression of genes related to tumor progression, including OPN, VEGF-c, snail, E-cadherin, and KLK2. This undoubtedly provides a new strategy for the treatment of prostate cancer [46]. In addition, the combination of GLP and immune checkpoint inhibitor anti-PD-1 monoclonal antibody can enhance the efficacy of anti-PD-1 immunotherapy. Therefore, GLP as a prebiotic also has the potential for use in tumor immunotherapy [47].

When it comes to in vivo studies, forty-eight five-week-old male C57BL/6 mice were randomly divided into four groups: control, model, GLPL (GLP low dose at 200 mg/kg), and GLPH (GLP high dose at 300 mg/kg). This research indicated that GLP reduced AOM/DSS-induced colitis and tumorigenesis. It improved AOM/DSS-induced microbial dysbiosis, increased the production of short-chain fatty acids, alleviated endotoxemia, and improved intestinal shielding function by inhibiting TLR4/MyD88/NF-κB signaling. These results suggested that GLP may be a promising prebiotic for the prevention or treatment of colorectal cancer [48]. In the research on a glucose-rich polysaccharide from *G. lucidum* (WSG) anti-lung cancer, twenty male C57BL/6 mice were randomly divided into two groups and treated with intraperitoneal injections of PBS and WSG (75 mg/kg) for 21 days, respectively. The results indicate that WSG can exert anti-cancer effects by reducing the phosphorylation levels of Akt, ERK1/2, FAK, and Smad2. Importantly, WSG can induce the degradation of epidermal growth factor (EGF) and transforming growth factor-β (TGF-β) receptors in lysosomes and proteasomes, respectively. In vivo experiments have also demonstrated their ability to inhibit lung tumor growth, reduce the volume of lung metastatic nodules, and improve the survival rate of lung cancer bearing mice [49]. Meanwhile, in another study, fifteen six-week-old C57BL/6 male mice were randomly divided into three groups and treated with PBS, WSG, and cisplatin, respectively, for 21 days. It is revealed that the co-treatment of GLP and cisplatin in xenograft mice carrying lung cancer exhibited synergistic inhibitory effects on tumor growth, nodular lung metastasis, and promotion of cell apoptosis. This discovery highlights the potential of GLP as a complementary or adjuvant compound in cancer treatment, significantly enhancing the therapeutic efficacy of cisplatin [50]. To further expand the scope, scholars have explored the anti-cancer properties of polysaccharides extracted from *Ganoderma* spore germ layer removed spores (RSGLP) and spore layer broken spores (BSGLP). In this study, RSGLP demonstrated superior efficacy in inhibiting the growth of HCT116 and NCI-H460 xenograft tumors and in reducing tumor-induced splenomegaly in nude mice. In this study, four-week-old male BALB/c nude mice were randomly assigned to control, model, and GLP treatment groups, namely, a low-dose RSGLP or BSGLP group (150 mg/kg) and a high-dose RSGLP or BSGLP group (300 mg/kg) by body weight (n = 8 per group). The results indicated that RSGLP shows a stronger ability to regulate immunity compared to BSGLP. Moreover, RSGLP exhibits excellent anti-inflammatory activities, such as regulating the secretion of inflammatory factors such as COX-2, IL-1 β, iNOS, and TNF-α. This significant performance difference highlights the potential of RSGLP as a novel anti-cancer agent with anti-tumor and immunostimulatory effects [51].

GLPs serve as multifunctional components in nanocomposite particles, contributing both structurally and biologically. These polysaccharides enhance the water dispersibility and stability of the superparamagnetic reduced graphene oxide (rGO-Fe_3_O_4_) nanoparticles by forming hydrogen bonds. This function is vital for maintaining uniform distribution and preventing the agglomeration of nanocomposites, which is essential for their use in drug delivery [52]. RCGDDH NPs, a rutin-carboxyphenyl boronic acid (CPBA)–GLP-dithiodipropionic acid (DPA)–dihydroartemisinin (DHA)/10-hydroxy camptothecin (HCPT) novel polymeric nanoparticle system, was developed using GLP as the base. These nanoparticles are dual-responsive to pH and redox conditions. The system is designed to release three anti-cancer drugs in a controlled manner: rutin is released in the acidic microenvironment of tumor tissues, while DHA and HCPT are released in the redox-rich environment of tumor cells. GLP, acting as the carrier, not only stabilizes the nanoparticle structure but also enhances its anti-cancer activity, making it an effective drug delivery platform for targeted cancer therapy [53]. A sandwich-structured anti-tumor medical film is designed with three distinct layers: the top and bottom layers made of ethyl cellulose (EC) embedded with *G. lucidum* triterpenoids (GLT), and a central layer made of polyvinyl alcohol (PVA) containing GLP. This innovative film harnesses the bioactive properties of both GLP and GLT, effectively inhibiting tumor cell invasion, limiting tumor cell proliferation, and promoting sustained drug release, making it a promising therapeutic tool in cancer treatment [54]. These approaches align well with the need for targeted, controlled release in cancer treatment, where the enhanced specificity offers a significant advantage over conventional chemotherapy.

Lucidumol A is a lanostane-type triterpene extracted from *G. lucidum*. Studies have shown that Lucidol A can inhibit the progression of colorectal cancer cells by inducing cytotoxicity and reducing metastatic ability while enhancing the anti-inflammatory capacity of the surrounding cells [55].

In clinical treatments for cancer, *G. lucidum* is used as an adjuvant therapy for chemotherapy or radiotherapy to prolong long-term survival, improve quality of life, and regulate immunity. In order to evaluate the role of *G. lucidum* in cancer treatment, multiple clinical studies have been published since 1997. Evidence suggests that after taking *G. lucidum*-related products such as Lingzhi capsules, *G. lucidum* extract, or *G. lucidum* spore powder supplements, there are changes in NK cell activity and CD4/CD8 ratio, indicating that cancer patients exhibit a series of cellular immune enhancements. In addition, giving *G. lucidum* extract can improve the quality of life of lung cancer patients. In addition, oral *G. lucidum* spore powder can also improve the physical health and fatigue subscale of breast cancer patients receiving endocrine therapy. However, there is still a lack of sufficient evidence to encourage the use of *G. lucidum* as a first-line treatment for cancer [56].

In summary, the collective results of these studies indicate that GLP has great potential in cancer management, with both direct cancer prevention and anti-cancer effects, as well as the potential to enhance the effectiveness of existing cancer treatments. These effects include a wide range of mechanisms, from inducing cell apoptosis to regulating the immune system and anti-inflammatory activity. It is crucial to conduct long-term research in the future to evaluate the impact of GLP on tumor progression, recurrence, and patient survival. Before widespread clinical implementation, it is necessary to rigorously evaluate the safety and toxicity characteristics of GLP, and comprehensive research should also be conducted to ensure its safety for human consumption. Furthermore, although GLP-based NP system studies have demonstrated the anti-tumor efficacy of these systems in vitro and in vivo, more comprehensive preclinical and clinical trials are needed to evaluate their safety and efficacy in human patients. Therefore, it is crucial to address potential challenges related to biocompatibility, stability, and long-term safety.

### 3.5. Lentinula edodes

#### 3.5.1. Occurrence and Ecology

*Lentinula edodes* (*L. edodes*) P, commonly known as shiitake—but also referred to as flower mushrooms, fragrant mushrooms, and fragrant truffles—naturally thrives in the damp, temperate climates of regions across North and South America, Asia, and Australia. They are a type of edible fungus and are often referred to by various accolades such as “King of Mountain Delicacies”, “Queen of Mushrooms”, and “Queen of Vegetables”. The fruiting bodies of shiitake mushrooms can be solitary, gregarious, or clustered. When immature, they exhibit a hemispherical shape, which flattens as they mature. The surface color of shiitake mushrooms ranges from light brown, beige, and dark brown to a deep cinnamon, reflecting their nature as a cold-loving and temperature-fluctuating mushroom species [57].

#### 3.5.2. Anti-Cancer Properties of Bioactive Components from *L. edodes*

*L. edodes* have garnered significant attention for their bioactive components that exhibit anti-tumor activity. These components include polysaccharides, glycoproteins, and peptides, which have been shown to exert direct cytotoxic effects on cancer cells, to modulate the immune system, and to influence the tumor microenvironment. Lentinan, the polysaccharide extracted from *L. edodes*, has been widely used in clinical practice as a cancer treatment adjuvant as it can improve the immunity function in the human being [58]. As an extensively studied compound, most of the foundational research has been established in recent decades. Consequently, recent publications have tended to focus on novel derivatives, structural modifications, or combination therapies involving lentinan rather than on lentinan alone. This trend reflects lentinan’s mature status in the field and ongoing efforts to enhance its clinical efficacy.

Immune checkpoint blockade (ICB) therapy has made significant progress in cancer treatment, but some cancers do not respond or develop resistance to ICB therapy, and patients may experience immune-related adverse events. Therefore, finding combination therapy strategies to improve the efficacy of ICB is of great significance. AHCC^®^ (Active Hexose Correlated Compound) is a standardized extract of *L. edodes* mycelia [59]. Compared to using DICB alone, Hong Jai Park et al. found that AHCC^®^-combined DICB treatment can significantly reduce the tumor volume of MC38 tumor mice. Research has shown that this significant therapeutic effect is closely related to the increase in cytotoxic molecules (such as perforin) and the proliferation ability (such as Ki-67 expression) of tumor-infiltrating CD8^+^ T cells. Meanwhile, AHCC^®^-combined DICB treatment also altered the composition of the mouse gut microbiome, increasing the abundance of *Ruminococcaceae* family bacteria, which hare associated with enhanced immune therapy efficacy.

Cortex protein (CTTN), an actin nucleation-promoting factor, has been reported to be up-regulated in pancreatic cancer cells and is associated with migration, invasion, and metastasis. A recent study has suggested that 10 mg/mL AHCC^®^ significantly inhibited the migration of gemcitabine-resistant pancreatic cancer cell line-KLM1-R cells. Also, the expression level of CTTN protein was significantly reduced in KLM1-R cells with AHCC^®^ treatment, while actin expression was not affected. The above results indicate that AHCC^®^ has anti-metastasis potential in pancreatic cancer cells [60].

Cortactin plays a crucial role in the migration, invasion, and metastasis of prostate cancer cells, and its overexpression is closely related to the malignant progression of cancer. Another study suggested that AHCC significantly down-regulated the expression of cortactin in both LNCaP.FGC and DU145 cells, but it was shown to be ineffective for highly metastatic PC-3 cells. This difference may be related to factors such as the malignancy of cells and androgen dependence [61].

The polysaccharides extracted from *L. edodes* could inhibit the proliferation of MCF-7 cells, which is related to the increasing expression of the p53 gene while suppressing the activity of the HER-3 gene in a dose- and time-dependent manner [13]. Moreover, in another study, *L. edodes* aqueous extracts inhibited the A549 cells, and inhibition effectiveness was closely related to the secondary metabolite indole-3-lactic acid (ILA) [62].

Comprehensive reviews confirm that the structure and biological activity of *L. edodes*-derived polysaccharides are highly dependent on extraction methods and chemical modifications, which alter molecular weight, monosaccharide composition, and branching [58]. Furthermore, selenium-enriched polysaccharides (Se-polysaccharides) from mushrooms are recognized for their synergistic anti-tumor and immunoregulatory activities, attributed to the combined effects of selenium and polysaccharides [63]. β-Glucan is an important active ingredient in shiitake mushroom extract. Studies have shown that β- glucan has a cytotoxic effect on breast cancer cells but has no cytotoxic effect on normal breast cells [64]. β-Glucan also reduced the secretion of IL-1β and IL-6 by LPS-activated THP-1/M cells. Higher concentrations of β-glucan affect the survival ability of tumor cells, induce strong anti-cancer immune responses, and inhibit the activity of topoisomerase I, which is considered an important target for cancer chemotherapy [65].

Furthermore, water-soluble glycoprotein fractions from the vegetative mycelium of *L. edodes* have been shown to inhibit the metabolic activity of various human and animal cancer cell lines, including A549 (human lung adenocarcinoma), HeLa (human cervical carcinoma), Hep-2 (laryngeal cancer), SPEV-2 (porcine kidney embryo), and C6 (rat glioma) cells. In this study, high-molecular-weight glycoproteins (60–250 kDa) exhibited the strongest cytotoxicity towards C6 cells, while low-molecular0weight glycoproteins (10–100 kDa) showed inhibition rates of up to 80–95% on all tested cell lines at high concentrations [66].

In addition to immune modulation, bioactive components from *L. edodes* can directly inhibit cancer cell growth and induce apoptosis. Latcripin-7A (LP-7A), a peptide derived from *L. edodes*, has been shown to induce apoptosis, autophagy, and cell cycle arrest in G1phase of human gastric cancer cells (SGC-7901 and BGC-823) by inhibiting the PI3K/Akt/mTOR signaling pathway [67].

Recent studies on selenium-enriched polysaccharides isolated from *L. edodes* mycelium (e.g., Se-Le-30) revealed context-dependent effects on T lymphocytes. Se-Le-30 modulated the expression of immune checkpoints (CD279/PD-1 and CD366/TIM-3), influenced T cell subpopulations, and stimulated cytokine production, including IL-6 and IL-10, indicating its immunomodulatory potential via both direct and indirect mechanisms [68].

Another study demonstrated that Se-Le-30 inhibited T cell proliferation when stimulated with anti-CD3 but enhanced proliferation when stimulated with anti-CD3/CD28. It modulated early activation markers (CD69, CD25) and regulated cytokine production (e.g., increasing IFN-γ, IL-6, and IL-10) depending on the activation context, further confirming its dual immunomodulatory behavior [69]. Additional evidence for the selective immunosuppressive activity of Se-Le-30 was provided in a study showing that this selenopolysaccharide significantly inhibits the proliferation of anti-CD3-stimulated PBMCs, reduces TNF-α production by CD3^+^ T cells, and decreases NK cell cytotoxicity. These effects suggest a non-typical immunoregulatory profile for a mushroom-derived polysaccharide and highlight the potential of Se-Le-30 in modulating excessive immune responses [70].

In a recent approach, *L. edodes* mycelium was cultured in media enriched with selenium and zinc ions, resulting in mycelial extracts with modified immunomodulatory activity. These dual-enriched preparations significantly influenced T cell activation, particularly by altering the expression of key activation markers such as CD25 and immune checkpoint receptor PD-1 on CD8^+^ T cells following stimulation with anti-CD3 or anti-CD3/CD28 antibodies. The results suggest that the content of selenium and zinc within the mycelium not only enhances its biological activity but may also fine-tune the immune response in a context-dependent manner. This points to a promising strategy for developing functional immune-nutritional supplements or supportive cancer therapies based on micronutrient-enriched mushroom biomass [71].

Selenium-containing exopolysaccharides from the post-culture medium of *L. edodes*, composed mainly of highly branched 1-6-α-mannans, also exhibited immunosuppressive properties, selectively inhibiting T cell proliferation and protecting normal cells from oxidative stress, though their activity was lower than that of Se-polyglucans isolated directly from the mycelium, likely due to differences in selenium binding and oxidation state [72].

In summary, *L. edodes* is particularly known for its polysaccharide, lentinan, which plays a critical role in enhancing immune responses. Unlike other mushrooms, lentinan has been extensively studied for its ability to stimulate the immune system, making it an essential part of cancer therapy and immune support. Lentinan, along with other bioactive compounds such as glycoproteins and peptides, is used in adjuvant cancer therapy. This distinguishes *L. edodes* from many other medicinal mushrooms as its polysaccharides have a documented history of improving the efficacy of chemotherapy and radiotherapy. Selenium-enriched *L. edodes* polysaccharides exhibit dual properties of anti-cancer and immunomodulatory activity, setting them apart from other species that may not have this unique enrichment. However, to better understand their anti-cancer mechanism, further research is needed focusing on elucidating the specific molecular mechanisms and identifying the most effective bioactive compounds for clinical application is required.

### 3.6. Laetiporus sulphureus

#### 3.6.1. Occurrence and Ecology

*Laetiporus sulphureus* (*L. sulphureus*), also known as sulfuric polypore or sulfur-colored polypore, is a fungus that naturally parasitizes a variety of trees, causing brown rot (decay and the formation of holes in wood), which leads to the rapid death of trees. It most commonly inhabits deciduous trees, particularly those belonging to the oak, locust, and poplar genera, and seldom dwells on conifers. The *L. sulphureus* is very common and is mainly found in North America and Europe, but it has also been reported in South America, Africa, Asia (including China, Laos, and Thailand), and Australia [73].

#### 3.6.2. Anti-Cancer Properties of Bioactive Components from *L. sulphureus*

Modern scientific research has suggested the potential anti-cancer properties of *L. sulphureus*, attributing these effects to its rich content of bioactive compounds such as polysaccharides, triterpenoids, and lectins. Their anti-cancer properties involve multiple pathways, inducing cell cycle arrest, apoptosis induction, and inhibition of cell migration.

Polysaccharides and their sulfated derivatives are among the most extensively studied bioactive components in *L. sulphureus* due to their diverse biological activities, including anti-cancer effects. Recent studies have demonstrated that sulfated polysaccharides (SPS) from *L. sulphureus* exhibit more potent anti-cancer activity compared to non-sulfated polysaccharides (PS) [74]. The anti-cancer mechanisms of SPS involve multiple pathways, including cell cycle arrest, apoptosis induction, and the inhibition of cell migration. Specifically, SPS have been shown to induce cell cycle arrest at the G0/G1 phase by down-regulating CDK4 and cyclin D1 while up-regulating p21 protein expression in triple-negative breast cancer cells (MDA-MB-231). This selective cytotoxicity towards cancer cells, without significant effects on normal cells, highlights the potential of SPS as a safe and effective anti-cancer agent. Additionally, SPS inhibit cancer cell migration, which is crucial for preventing metastasis [74,75].

Lectins, another class of bioactive compounds in *L. sulphureus*, have also attracted attention for their anti-cancer potential. A recent study using zebrafish xenograft models demonstrated that *L. sulphureus* lectin (LSL) effectively inhibited the growth, neovascularization, and metastasis of human colorectal cancer cells (HCT-116) and mouse melanoma cells (B16-F10). LSL can significantly inhibit the development and metastasis of colorectal cancer and melanoma, without causing toxicity to normal tissues, organs, and formed blood vessels with little effect on neutrophils. LSE did not exhibit anti-cancer activity at high doses (200 μg/mL) but it can also inhibit the angiogenesis and metastasis of rectal cancer and melanoma, without causing significant changes in neutrophils. Moreover, the anti-angiogenic activity of LSL is significantly higher than that of LSE, and it is comparable to the clinical drug sunitinib in inhibiting the angiogenesis of ISVs and SIVs. Moreover, its inhibitory effect is superior to sunitinib at the dose of 50–100 μg/mL. Therefore, LSE deserves further in-depth research on the specific molecular mechanisms underpinning its anti-cancer activity [76].

Triterpenes are another group of bioactive compounds with anti-cancer properties in *L. sulphureus*. Recent studies have identified two new triterpenoids, laetiporins C and D, from the fruiting bodies of *L. sulphureus*. They all exhibit anti-proliferative activity with respect to MCF-7 cells, indicating their potential in inhibiting tumor cell growth. The anti-cancer mechanisms of triterpenoids generally involve cell cycle regulation, apoptosis induction, and the inhibition of angiogenesis and metastasis, similar to those of polysaccharides and lectins, which require further exploration [77].

However, compared with *G. lucidum* and other well-studied fungi, the research on anti-cancer polysaccharides, triterpenoids, and other compounds in *L. sulphureusis* is less systematic. In contrast to *L. sulphureusis*, *G. lucidum* has been extensively investigated, with multiple clinical trials confirming its anti-cancer effects. Most studies on *L. sulphureusis* have focused on in vitro experiments and animal models, and there is a lack of clinical data, which limits the evaluation of the accuracy and effectiveness of *L. sulphureusis*’s anti-cancer effects in humans. In addition, existing research has primarily targeted common cancers such as breast, colorectal, and liver cancers, with relatively little attention being paid to rare yet severe cancers like pancreatic cancer and cholangiocarcinoma. This makes it difficult to comprehensively assess the universal and cancer-specific anti-cancer effects of *L. sulphureusis*’s active components.

### 3.7. Boletus edulis

#### 3.7.1. Occurrence and Ecology

*Boletus edulis* (*B. edulis*) belongs to the *Boletaceae* family and the *Boletus* genus of fungi. The cap diameter ranges from 8 to 25 cm, being hemispherical to convex-mirror-shaped, often with depressions on the surface, initially cinnamon to dark cinnamon in color, and becoming lighter as it matures. *B. edulis* is mainly distributed across Europe (including Italy, France, Switzerland, Germany), as well as in North America, Japan, China, Australia. It typically grows on the ground in coniferous forests or mixed coniferous and broadleaf forests during the summer and autumn seasons, and it sometimes also grows in open spaces on the forest edge and roadsides. This mycorrhizal fungus exhibits selective specificity for the plants it parasitizes, forming symbiotic relationships with higher plants and demonstrating a strong preference for its host species [33].

#### 3.7.2. Anti-Cancer Properties of Bioactive Components from *B. edulis*

Beyond its gastronomic appeal, B. edulis has garnered significant attention for its potential anti-cancer properties. Extensive research has elucidated various mechanisms through which bioactive components from B. edulis exert anti-tumor effects, including by inducing apoptosis, cell cycle arrest, and by regulating autophagy-related proteins, which increases the ROS level, offering promising insights into its application in cancer therapy.

One of the key mechanisms by which *B. edulis* combats cancer is through inducing cell cycle arrest. A new anti-tumor protein (designated as *B. edulis* or in short BEAP) has been isolated from the dried fruiting body of edible *B. edulis*, a novel multifunctional protein with anti-tumor and anti-metastatic abilities. Research has shown that BEAP exhibits strong anti-cancer activity against A549 cells in vitro and in vivo. BEAP inhibits the proliferation of lung-cancer-cell-line A549 by inducing apoptosis through increasing the Bax/Bcl-2 ratio, promoting the release of cytochrome C and activating caspase-3 and caspase-9. Moreover, BEAP induced cell cycle arrest in the G1 phase by reducing the related protein expression of E2F and CDK4. In addition, BEAP also suppressed cell migration effectively and inhibited the migration-related protein expression, such as that of vinculin. An in vivo study indicated that BEAP significantly inhibited the growth of A549 solid tumors in mice. It is a novel multifunctional protein with anti-tumor and anti-metastatic abilities [12].

Another study indicated that BEAP has the potential to regulate several kinds of effects. BEAP induced apoptosis in A549 cells by activating the MAPK signaling pathway, accompanied by a significant decrease in the expression of the negative regulatory proteins Alix and caspase-9. In addition, the expression of autophagy-related proteins P62 and LC3 II was up-regulated in A549 cells after treatment with BEAP, showing concentration- and time-dependent effects, indicating that BEAP can continuously activate the autophagy pathway in A549 cells. Further experiments have shown that BEAP could activate autophagy through the mTOR/AMPK pathway and damage-regulated autophagy regulator DRAM. By using appropriate autophagy inhibitors, such as Baf-A1, the anti-tumor effect of BEAP on A549 cells could be enhanced, BEAP-induced apoptosis of A549 cells was enhanced, and the degree of cell arrest in the G1 phase was increased, further inhibiting the malignant proliferation of A549 cells. This indicates that the combination of autophagy inhibitors and anti-tumor drugs has practical application value [78].

A novel acidic cold water-soluble polysaccharide (BEP) containing alpha and beta glycosidic bonds was isolated from *B. edulis*. Research has shown that BEP can inhibit the proliferation and induce the apoptosis of MDA-MB-231 and Ca761 cancer cells through the mitochondrial pathway. Additionally, it can induce cell arrest in the S phase and G0/G1 phase. Western blot results suggest that BEP can increase the ratio of Bax/Bcl-2 in MDA-MB-231 and Ca761 cells, promote the release of cytochrome C, and activate the expression of caspase-3 and caspase-3 [79].

BE extract is mainly composed of disaccharides (mainly trehalose), phenolic compounds (such as taxifolin, rutin, and ellagic acid), and minerals (such as K, P, Mg, Na, Ca, Zn, and Se). Research has shown that BE extract reduces proliferation by inducing G0/G1 phase cell cycle arrest and inducing cell death through autophagy and apoptosis, changes in mitochondrial membrane potential, and the activation of caspase-3 in the Caco-2 cell line. BE extract alters cellular redox balance by increasing ROS levels and alleviates Caco-2 inflammation by reducing iNOS and COX-2 mRNA expression, as well as COX-2 protein expression. BE extract also inhibits the oxidative stress level caused by H_2_O_2_ and protects the intestine, but its exact mechanism still needs further verification through in vivo experiments [80].

Although existing research suggests that the active substances of *B. edulis* have anti-cancer potential, further in-depth study of the mechanism of *B. edulis* is still needed to explore other possible cell death modes and their interactions with apoptosis, apoptosis, and autophagy, providing a theoretical basis for the development of more effective anti-cancer strategies and drugs. In addition, the combined effects of BEAP and other drugs can be studied to find better treatment options.

### 3.8. Phellinus igniarius

#### 3.8.1. Occurrence and Ecology

*Phellinus igniarius* (*P. igniarius*) is an inedible mushroom species that lacks a distinct flavor. It is widely distributed across North America, Europe, and Asia, typically found in riverbank forests and along streams. This species primarily grows on living willow trees and, less frequently, on trees of the elm genus. *P. igniarius* is a perennial fungus with a lifespan of 10 to 15 years, and it is known to cause white rot in wood. The young fruiting bodies appear nodular and spherical, but they later become flat and convex, with a grayish-black surface that darkens over time and may develop cracks. The cap’s margin is broad and rounded, and the fruiting body grows laterally from the tree trunk. The tubular membrane shell is multi-layered, and the 5 mm thick tubes share the same color as the fruiting body. The flesh of the fruiting body is woody, fibrous, and deep reddish-brown in color [81].

#### 3.8.2. Anti-Cancer Properties of Bioactive Components from *P. igniarius*

According to a recent study, *P. igniarius* extract can transform gastric cancer cells (SGC-7901) from flat to spherical and improve the height and surface roughness of the cells. The adhesion force of cells decreases, and the elastic modulus increases. However, there is no significant difference in the morphology and mechanical properties of gastric epithelial cells (GES-1). Also, *P. igniarius* extract has a high cytotoxic effect on gastric cancer cells, but its toxic side-effects on gastric epithelial cells are relatively small [82].

It has been reported that the water-soluble intracellular polysaccharide IPSW-1, extracted from the mycelium of the medicinal fungus *P. igniarius*, inhibits HepG2 cell proliferation in a dose-dependent manner. IPSW-1 also significantly suppresses the migration and invasion ability of HepG2 cells and reduces the expression of MMP-7 and RhoA proteins. Moreover, IPSW-1 inhibits the degradation of autophagosomes, leading to the accumulation of LC3II protein and affecting autophagic flow. In addition, IPSW-1 reduces mitochondrial membrane potential, increases the expression of Bax and cleaved caspase-3 proteins, and decreases the expression of Bcl-2 protein, thereby inducing apoptosis in HepG2 cells [83]. Another study showed that PIP (*P. igniarius* polysaccharide) can dose-dependently inhibit HepG2 cell proliferation. PIP can not only induce cell cycle arrest but also promote HepG2 cell apoptosis by increasing ROS levels, activating the AKT/p53 signaling pathway, inducing mitochondrial release of cytochrome c, and activating caspase-3. Further, WB experiments showed that the expression of p-AKT and Bcl-2 decreased, while the expression of p53, cytochrome c, Bax, and cleaved caspase-3 increased [84].

Osmundacetone (OSC) is a bioactive phenolic compound isolated from *P. igniarius* that could effectively inhibit cell proliferation and induce the G2/M phase cell cycle arrest of H460 and A549 lung cancer cells. In vivo experiments have shown that OSC significantly inhibits the growth of transplanted tumors and has no significant effect on the body weight and major organs of mice at experimental doses. Molecular mechanisms indicate that OSC down-regulates the expression of GLUD1, affects the glutamine/glutamate/–KG metabolic axis and mitochondrial oxidative phosphorylation, and inhibits the proliferation and tumor growth of lung cancer cells [85].

## 4. Conclusions and Perspective

### 4.1. Conclusions

Due to the rising incidence rate and mortality rate of cancer, as well as the heavy economic and psychological burden it has brought to society and patients, cancer is still a public health event of major concern in today’s society. Modern cancer treatment methods, including surgery, radiotherapy, chemotherapy, immunotherapy, and targeted therapy, have disadvantages such as high cost, obvious side-effects, and easy drug resistance. Scientists are committed to finding efficient and low-toxicity alternative medical methods.

**Figure 3:**
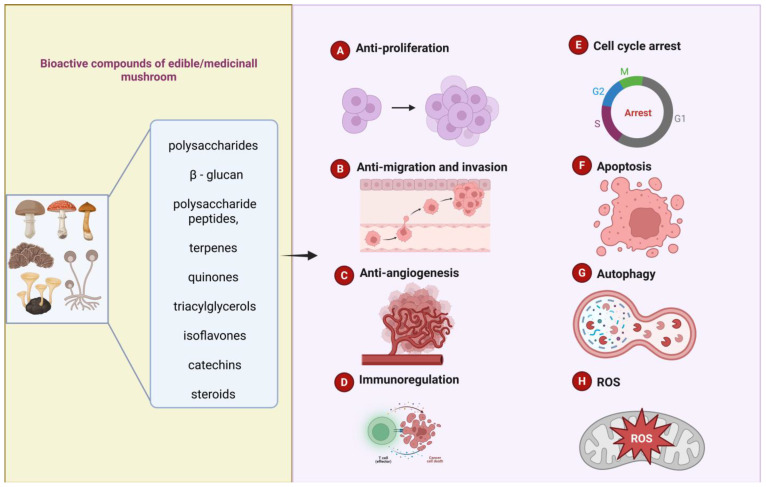
Anti-cancer mechanism of bioactive compounds in edible/medicinal mushrooms. This figure was created in Biorender (https://www.biorender.com/).

**Figure 4:**
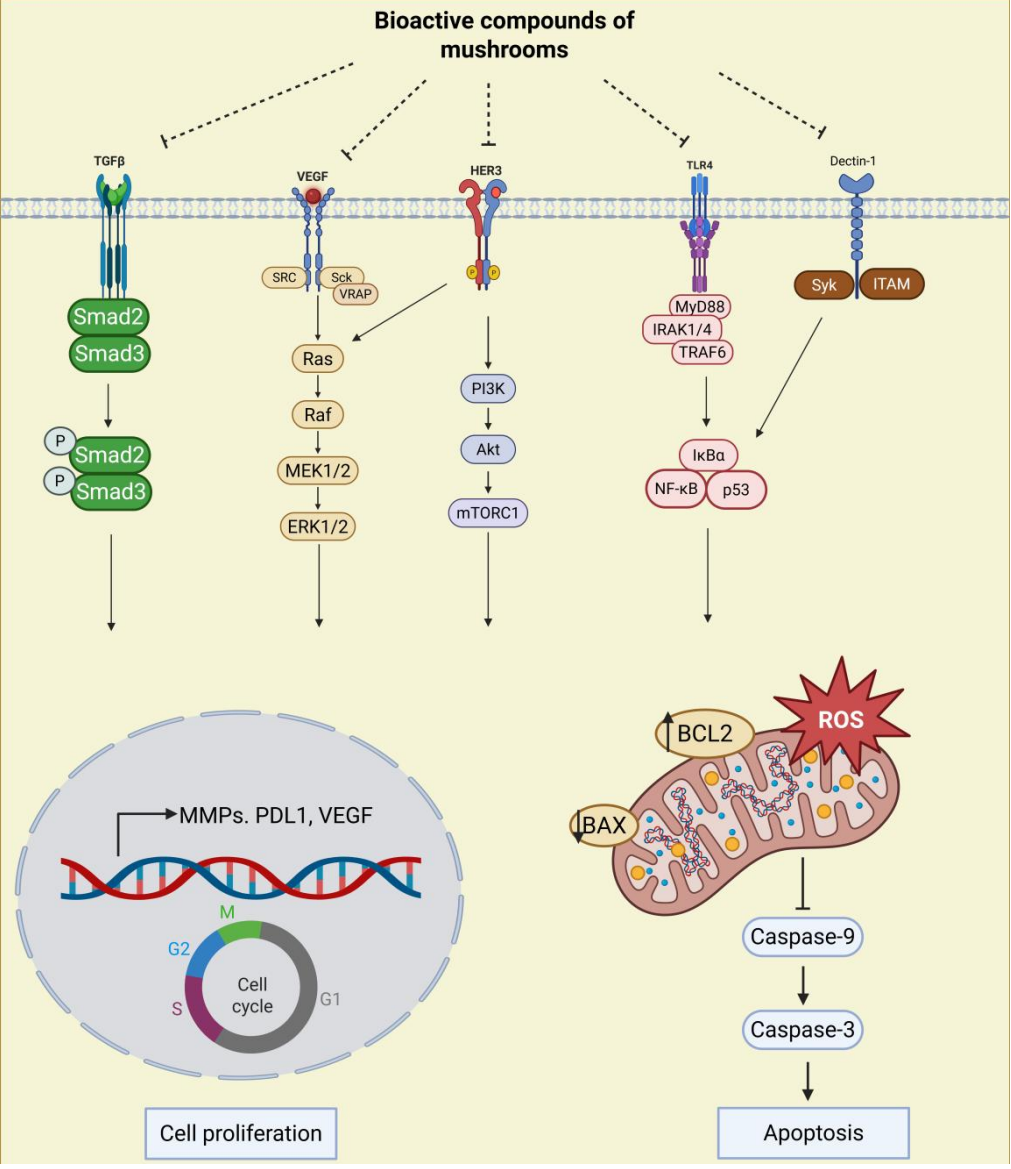
Signaling pathways regulated by bioactive compounds of edible/medicinal mushrooms. This figure was created in Biorender (https://www.biorender.com/).

Exploring compounds with anti-cancer potential in natural products has been a focus of attention for pharmaceutical scientists in recent decades. Many researchers have favored mushrooms in recent years due to their high content of active substances. We have summarized the common edible/medicinal mushrooms introduced to this discussion in the past five years, including *S. commune*, *T. versicolor*, *G. frondosa*, *G. lucidum*, *L. edodes*, *L. sulphureus*, *B. edulis*, and *P. igniarius,* as well as their direct and/or indirect anti-cancer effects and the mechanisms involved (Table 1 and Figure 3 and Figure 4). The bioactive compounds, including polysaccharides (especially β-glucan), polysaccharide peptides, glycoprotein fractions, triterpene, etc., found in edible and medicinal mushrooms have considerable potential as both functional foods and dietary supplements, with promising applications in cancer prevention and treatment. Mushrooms like *G. lucidum* and *L. edodes* are consumed for their health benefits in some Asian countries, while their extracts are increasingly used in nutritional supplements. These bioactive compounds can inhibit cancer cell proliferation, migration, invasion, and angiogenesis in vivo and in vitro, which may be related to mechanisms such as activating immune function, inducing cell cycle arrest, apoptosis, and autophagy. Some studies involve the regulation of pathway mechanisms, including the PI3K/AKT signaling pathway, the EGFR/Raf signaling pathway, the mTOR/AMPK signaling pathway, the P53 signaling pathway, the Dectin-1/Syk/NF-κB signaling pathway, and the TLR4/NF/κ B signaling pathway. Moreover, the mechanisms through which these compounds exert anti-cancer and immune-modulating effects suggest that in the future, these mushrooms, or their bioactive components, could be developed into pharmaceutical drugs for targeted cancer therapies. Therefore, mushrooms present a valuable dual role as both food sources and potential therapeutic agents.

This review has certain limitations as some mushroom species with possible or evaluated anti-tumor activities, such as *Agaricus subcultens*, were not included in this review. In the future, we will continue to focus on mushroom active substances with anti-cancer potential beyond the scope of this study, providing a theoretical basis for clinical drug development.

### 4.2. Perspective

Although there have been numerous studies on the direct or indirect anti-cancer effects of mushroom-bioactive compounds, these studies have had significant limitations. For example, most of these studies were conducted via in vitro experiments, and often, the experimental design was too simple and lacked quality control, which made the experimental results somewhat questionable. Moreover, there is still a serious lack of related in vivo experiments and large-scale, standardized, and high-quality clinical studies. In addition, current research is still focused on verifying the anti-cancer effects; thus, the molecular mechanisms involved have not been thoroughly and comprehensively elucidated. Furthermore, there is still a lack of effective and widely applicable methods for extracting and identifying active substances from mushrooms. All of these factors may hinder the development of mushroom active ingredients as clinical anti-cancer candidate drugs to varying degrees.

Therefore, in future research on bioactive in mushrooms, we should strive to explore more efficient and accurate extraction and purification methods. On the one hand, existing advanced technologies such as ultrasonic extraction and enzyme extraction can be used for reference [87]. On the other hand, the combination of advanced chromatographic technologies, such as gel filtration chromatography and ion exchange chromatography, can more effectively separate and purify the bioactive compound in mushrooms [88]. Multiple analytical methods, including atomic absorption spectroscopy (AA), can also be considered to comprehensively and deeply determine the effective bioactive compounds in mushrooms [89], laying a solid foundation for subsequent research.

For in vitro experiments, the research and application of new drug delivery systems provide new ideas for improving the anti-cancer effect of mushroom bioactive compounds [90]. We should actively explore the application of nano delivery materials in the delivery of mushroom active ingredients, as well as optimizing the preparation process and the performance of delivery systems, in order to achieve better therapeutic effects.

For in vivo experiments, in addition to conventional experimental design and operation, emphasis should also be placed on exploring bioavailability. By incorporating bioavailability research into the experimental design, it is possible to gain a deeper understanding of the changes in mushroom active ingredients during digestion and absorption, providing important references for optimizing dosing regimens and developing formulations [91,92].

More importantly, large-scale, standardized, and multicenter clinical studies are a key step in evaluating the anti-cancer effects of mushroom active substances [93]. This research design can fully consider individual differences and clinical realities, improving the reliability and universality of the research results. We should actively organize and participate in such clinical studies, strictly follow scientific standards for operation and statistical data analysis, comprehensively and objectively evaluate the direct or indirect anti-cancer effects of mushroom active ingredients in the human body, and provide strong evidence for promoting the application of mushroom active ingredients in clinical tumor treatment.

Through the aforementioned efforts, we have reason to believe that the extraction, purification, and identification techniques for bioactive compounds in edible/medicinal mushrooms will be significantly improved, and their anti-cancer efficacy and mechanisms of action will be more deeply and comprehensively revealed, thus opening up broader prospects for the development and utilization of mushrooms in the pharmaceutical field.

## Figures and Tables

**Table 1:** Summary of the anti-cancer research on the active ingredients of edible/medicinal mushrooms.

Mushroom	Bioctive Compounds	Effect	Pathways	Targets	Ref.
*S. commune*	SCFP (fruiting body polysaccharide)	Anti-glioma effect; immunomodulation	PI3K/Akt	↑Apoptosis	[19]
*S. commune*	SCP-1 (mucomannan)	Anti-lung cancer	PI3K/Akt/mTOR, Bax/Bcl-2	Cell cycle arrest (S phase), ↑Apoptosis	[10]
*S. commune*	Gold nanoparticles (AuNPs)	Anti-lung cancer	ROS-mediated apoptosis	↑ROS, ↓Cell viability (A549 cells)	[21]
*S. commune*	SPG (schizophyllan, β-glucan)	Immunomodulatory, drug delivery aid	Dectin-1/NF-κB	↑Cytokines, ↑Macrophage and NK cell activity	[22]
*S. commune*	Mixed extract (terpenoids, flavonoids, alkaloids)	Anti-proliferative	Not specified	HeLa, MCF-7, T47D, WiDr cell inhibition	[20]
*T. versicolor*	PSK, PSP	Broad-spectrum anti-tumor activity	EGFR, PD-L1	↓EGFR, ↓PD-L1, ↓STAT3, ↓p-STAT, ↓c-Jun	[23,25]
*T. versicolor*	PSP	Anti-colorectal cancer	EGFR signaling pathway	EGFR↓, c-Jun↓, STAT3↓, p-STAT↓, PD-L1↓,‘ apoptosis↑	[11]
*T. versicolor*	Musarin (12 kDa peptide)	Anti-CSC in colorectal cancer	EGFR-Raf signaling pathway	↓EGFR phosphorylation, CSC marker inhibition	[27]
*T. versicolor*	Mycelium ethanol extract	Anti-melanoma	Autophagy/apoptosis pathways	↑LC3-II, ↑MHC-II, ↑PD-L1, ↑PARP cleavage, ↓Migration	[26]
*T. versicolor*	Steroids (compound 1–5)	Anti-lung, colon, melanoma cancer	Not specified	Broad cytotoxicity on cancer cell lines	[28]
*G. frondosa*	GFPBW1 (β-glucan)	Anti-melanoma, adjuvant effect	Dectin-1/Syk/NF-κB signaling pathway	↑NF-κB; macrophage activation	[14]
*G. frondosa*	PPC (GFG-4)	Anti-liver cancer	TLR4/NF/κB signaling pathway, gut microbiota modulation	↑*Muribaculaceae*, *Bacillus*; ↓Lactic acid bacteria	[15]
*G. frondosa*	GFP-A	Immunomodulation Tumor inhibition	Not specified	↑TNF-α, IL-2, IFN-γ; ↑NK cell, macrophage and lymphocyte activity	[35]
*G. frondosa*	GFP1	Anti-lung cancer	P53/NF/κB signaling pathway, oxidative stress/apoptosis	↑ROS, ↓MMP, ↑Caspase-3/8/9, ↑p65 phosphorylation	[36]
*G. frondosa*	Se-LMW-GFP	Anti-gastric cancer	Mitochondrial and death receptor	↑Apoptosis proteins, ↑ROS, ↓MMP, cell cycle arrest in G1	[37]
*G.lucidum*	GLPS (polysaccharides)	Anti-OSCC (oral squamous cell carcinoma)	CSC/EMT signaling	↓CSC markers, ↓EMT markers	[42]
*G.lucidum*	FYGL (proteoglycan)	Anti-pancreatic cancer	ROS/autophagy	↑ROS, ↓autophagy	[45]
*G. lucidum*	GLP + flutamide/docetaxel	Enhanced prostate cancer therapy	Multiple	↓OPN, VEGF-c, Snail, E-cadherin, KLK2	[46]
*G.lucidum*	GLP	Anti-colorectal cancer	TLR4/MyD88/NF-κB	↓TLR4, ↓NF-κB, ↑SCFA, ↓endotoxemia	[48]
*G.lucidum*	GLP + anti-PD-1	Enhanced immunotherapy	Immune checkpoint modulation	↑anti-PD-1 efficacy	[47]
*G.lucidum*	GLP	Anti-lung cancer	Akt/ERK1/2/FAK/Smad2	↓phosphorylation, ↓EGFR and TGF-β receptors (lysosomal/proteasomal degradation)	[49]
*G.lucidum*	GLP + cisplatin	Synergistic lung cancer inhibition	Not specified	↑apoptosis, ↓metastasis	[50]
*G.lucidum*	RSGLP vs. BSGLP	Anti-breast/colon/liver/lung cancers	Anti-inflammatory	↓COX-2, IL-1β, iNOS, TNF-α	[51]
*G. lucidum*	Lucidumol A	Anti-colorectal cancer	Inflammation	COX-2↓, iNOS↓	[55]
*G. lucidum*	GLE (extract)	Targeted drug delivery	Not specified	Cytotoxic to A549, carrier for rGO-Fe₃O₄/Quercetin	[52]
*G.lucidum*	GLP (RCGDDH NPs)	Anti-tumor nano-delivery system	MMP-9 inhibition	↓MMP-9, dual pH/redox-responsive delivery	[53]
*G. lucidum*	GLT + GLP (film system	In vitro anti-cancer activity	Not specified	Effective against SGC-7901, A549, Hela, Caco-2	[54]
*G. lucidum*	GL extract/spore products	Clinical adjuvant (lung/breast cancer)	Immunomodulation	↑NK cells, ↑CD4/CD8 ratio, ↑QoL	[56]
*L. edodes*	AHCC^®^ (mycelial extract)	Enhanced ICB therapy (anti-tumor effect)	Immunomodulation, microbiome	↑Perforin, ↑Ki-67 (CD8^+^ T cells), ↑*Ruminococcaceae*	[59]
*L. edodes*	AHCC^®^	Anti-metastatic (pancreatic cancer)	Migration/invasion suppression	↓Cortactin (CTTN), no change in actin	[60]
*L. edodes*	AHCC^®^	Anti-metastatic (prostate cancer)	Migration/invasion suppression	↓Cortactin (LNCaP.FGC, DU145), no effect in PC-3	[61]
*L. edodes*	Latcripin-7A (LP-7A, peptide)	Anti-gastric cancer	PI3K/Akt/mTOR signaling	↑Apoptosis, ↑Autophagy, G1 arrest	[67]
*L. edodes*	Glycoprotein fractions	Cytotoxicity toward cancer cell	Not specified	↓Metabolic activity in A549, HeLa, Hep-2, SPEV-2, C6 cells	[66]
*L. edodes*	Polysaccharides	Anti-breast cancer	p53/HER-3 signaling	↑p53, ↓HER-3	[13]
*L. edodes*	Aqueous extract + ILA	Anti-lung cancer	Not specified	Inhibition of A549 cells (via indole-3-lactic acid)	[62]
*L. edodes*	β-glucan	Anti-breast cancer	Immune modulation	↓IL-1β, ↓IL-6, inhibition of topoisomerase I	[64]
*T. versicolor*	SMCV	Anti-glioblstoma	Anti-cancer effect and anti-invasive ability of T98G cells	↓TNF-α, ↓MMP3	[30]
*T. versicolor*	Polysaccharides	Enhance the function of RAW 264.7 macrophages	Immune modulation	↑iNOS, ↑TNF-α	[86]
*T. versicolor*	PBP	Tumor microenvironment modulation	Macrophage polarization (M2 → M1)	↑IL-6, ↑TNF-α, ↓IL-10, ↓TGF-β	[31]
*L. sulphureus*	Sulfated polysaccharides (SPS)	Anti-breast cancer	Cell cycle arrest, apoptosis	↓CDK4, ↓Cyclin D1, ↑p21, G0/G1 arrest	[74,75]
*L. sulphureus*	Lectin (LSL)	Anti-colorectal cancer, anti-melanoma	Anti-angiogenesis, anti-metastasis	↓VEGF, ↓neovascularization, comparable to sunitinib, no effect on neutrophils	[76]
*L. sulphureus*	Triterpenoids (laetiporins C and D)	Anti-breast cancer	Cell proliferation inhibition	Not specified; general antiproliferative activity on MCF-7	[77]
*B. edulis*	BEAP (boletus edulis anti-tumor protein)	Anti-lung cancer, anti-metastatic, induces apoptosis, G1 arrest, enhances autophagy	MAPK, mTOR/AMPK, Bax/Bcl-2, caspase-3/-9, DRAM	Bax↑, Bcl-2↓, Cytochrome c↑, Caspase-3/9↑, CDK4↓, E2F↓, Vinculin↓, LC3-II↑, P62↑, Alix↓	[12]
*B. edulis*	BEP (boletus edulis polysaccharide) acidic cold-water soluble polysaccharide	Anti-lung cancer, induces apoptosis, S and G0/G1 arrest, liver cancer	Mitochondrial apoptosis pathway	↑Bax/Bcl-2, ↑cytochrome c, ↑caspase-3, cell cycle arrest (S, G0/G1)	[78]
*B. edulis*	BE extract (trehalose, phenolic compounds, minerals)	Anti-colon cancer, induces apoptosis and autophagy, G0/G1 arrest, antioxidant, anti-inflammatory	ROS signaling, apoptosis, autophagy	↑ROS, ↓COX-2, ↓iNOS, ↑caspase-3, ↓MMP,	[79]
*P. igniarius*	IPSW-1 (intracellular polysaccharide)	Anti-liver cancer (HepG2)	Autophagy and Apoptosis-related pathways	↓MMP-7, ↓RhoA, ↑LC3-II, ↓Bcl-2, ↑Bax, ↑Cleaved caspase-3	[83]
*P. igniarius*	PIP (polysaccharide)	Anti-liver cancer (HepG2)	AKT/p53 signaling pathway	↓p-AKT, ↓Bcl-2, ↑p53, ↑Cytochrome c, ↑Bax, ↑Cleaved caspase-3	[84]
*P. igniarius*	Osmundacetone (OSC)	Anti-lung cancer (H460, A549)	GLUD1/glutamine metabolism axis	↓GLUD1, inhibition of glutamine/glutamate/α-KG axis, ↓OXPHOS	[85]

Abbreviations: *S. commune*, *Schizophyllum commune*; *T. versicolor*, *Trametes versicolor*; *G. frondosa*, *Grifola frondosa*; *G.lucidum*, *Ganoderma lucidum*; *L. edodes*, *Lentinula edodes*; *L. sulphureus*, *Laetiporus sulphureus*; *B. edulis*, *Boletus edulis*; *P. igniarius*, *Phellinus igniarius*. ↑—increase; ↓—decrease.
